# Social-Ecological Factors Associated With Higher Levels of Resilience in Children and Youth After Disaster: The Importance of Caregiver and Peer Support

**DOI:** 10.3389/fpubh.2021.682634

**Published:** 2021-07-29

**Authors:** Caroline McDonald-Harker, Julie L. Drolet, Anika Sehgal, Matthew R. G. Brown, Peter H. Silverstone, Pamela Brett-MacLean, Vincent I. O. Agyapong

**Affiliations:** ^1^Department of Sociology & Anthropology, Mount Royal University, Calgary, AB, Canada; ^2^Faculty of Social Work, University of Calgary, Calgary, AB, Canada; ^3^Department of Community Health Sciences, University of Calgary, Calgary, AB, Canada; ^4^Department of Computing Science, University of Alberta, Edmonton, AB, Canada; ^5^Department of Psychiatry, University of Alberta, Edmonton, AB, Canada

**Keywords:** disaster, children, youth, mental health, resilience

## Abstract

Children and youth are among the most vulnerable to the devastating effects of disaster due to the physical, cognitive, and social factors related to their developmental life stage. Yet children and youth also have the capacity to be resilient and act as powerful catalysts for change in their own lives and wider communities following disaster. Specific factors that contribute to resilience in children and youth, however, remain relatively unexplored. This article examines factors associated with high levels of resilience in 100 children and youth aged 5- to 18-years old who experienced the 2016 Fort McMurray, Alberta wildfire. A mixed-methods design was employed combining quantitative and qualitative data. Quantitative data was obtained from the Children and Youth Resilience Measure (CYRM-28) which measured individual, caregiver, and context factors influencing resilience processes among the participants. Qualitative data was collected through semi-structured interviews to gain further insight into the disaster experiences of children and youth. Quantitative findings reveal higher than average levels of resilience among the participants compared to normative scores. Qualitative findings suggest high levels of resilience were associated with both caregiver factors (specifically physical caregiving), and individual factors (primarily peer support). We discuss how physical caregiving and peer support during and after the wildfire helped mitigate the negative effects of disaster, thus bolstering children and youth's resilience. Implications for understanding the specific social-ecological factors that facilitate and support resiliency processes and overall recovery of children and youth following disaster are also discussed.

## Introduction

Resilience among children and youth following traumatic life events is increasingly being examined in various disciplinary fields, including psychiatry, social work, psychology, and sociology. Traumatic life events include, but are not limited to, abuse, death, war and conflict, health pandemics, and natural disasters. Natural disasters, including hurricanes, tornadoes, tsunamis, floods, and wildfires are increasing in both frequency and severity, often exacerbated by population growth, environmental degradation, and changes in global climate systems (1). Experiencing a natural disaster produces significant trauma for children and youth given the wide range of stressors involved, including “threat to one's own life and physical integrity, exposure to death and dying, bereavement, profound loss, social and community disruption, and ongoing hardship” (2). Due to their developmental life stage, dependence on adults, and limited access to child/youth-centered resources post-disaster, children and youth often are vulnerable to the devastating effects of disaster (3). After experiencing disaster, children and youth often experience increased behavioral problems, including insomnia, anxiety, depression, and post-traumatic stress disorder (PTSD) (2, 4–9).

The limited literature that has examined the experiences of children and youth post-disaster has largely focused on risk factors associated with negative outcomes rather than protective factors, resulting in a deficit-based approach (10–12). While understanding the risk factors and vulnerabilities of children and youth post-disaster is important, the need to understand the protective factors and strengths they possess is equally pressing as they can serve to mediate resiliency processes following the adverse experience of disaster (13, 14). Resilience is increasingly being viewed as involving not only individual characteristics, but also broader social-ecological factors such as family, peer, and community factors (13–16). Recent research which focuses on protective factors reveals that children and youth have the capacity to demonstrate resilience and act as powerful catalysts for change, recovery, and rebuilding within their families and communities following a disaster (17–19). This recent focus on resilience in disaster research is particularly relevant given that disasters are expected to rise globally in coming years due to the effects of climate change (20). While child and youth resilience research within the context of disasters has made significant gains, little is known about the specific factors that contribute to resilience in children and youth, and effective ways to support their overall health and well-being. Moreover, few studies have explored child and youth resilience from their own experiences, perspectives, and voices (11, 14, 17, 18, 21).

This article fills an important gap in the literature by exploring the lived experiences and voices of children and youth who experienced the 2016 Fort McMurray wildfire. The following analyses explores various factors associated with high levels of resilience among children and youth post-wildfire, including individual, caregiver, and context factors. Findings reveal that high levels of resilience among children and youth were associated with caregiver factors, specifically physical caregiving, and individual factors, namely peer support. This article discusses the implications these findings have for better understanding the social-ecological factors that facilitate and support the resiliency processes of children and youth post-disaster.

### The 2016 Fort McMurray Wildfire

Fort McMurray, Alberta is located in the Regional Municipality of Wood Buffalo (RMWB) in the Northeastern area of Alberta. Fort McMurray is home to numerous multi-nationally owned oil-sands projects directed by various oil and gas companies. Due to the resource-based industry of Fort McMurray, 35% of the population at the time of the wildfire was non-permanent/transient, referred to as the “shadow population” (22). The majority of this population resides within the active work camps near oilsands project sites. The gender distribution of the population is male-dominated, consisting of 55.4% males compared to 44.6% females. The population between the ages of 25 and 44 account for 43% of the total population, with the highest concentration (25%) being 25- to 34-years of age. The proportion of children and young adolescents 0- to 19-years of age constitute 23.9% of the overall population (22). On May 1st, 2016, the town of Fort McMurray experienced a devastating major wildfire coined “The Beast” (23). Over 88,000 people were evacuated as the wildfire threatened to engulf their homes and communities (23). Within just days, the wildfire spread across 590,000 hectares of land before firefighters and emergency crews could contain it (24). It is estimated that the wildfire caused $3.6 billion Canadian dollars in insured losses, the most catastrophic and costly disaster in Canadian history (25). Homes, schools, daycares, businesses, and entire communities were destroyed due to the wildfire. In addition to ongoing rebuilding and restoration efforts, individuals are continuing to recover from the many social emotional, and psychological impacts of the wildfire. Children and youth were particularly affected by the wildfire as the effects of both evacuation and destruction from the wildfire persisted for an extended duration of time, disrupting children and youth's day-to-day routines, functioning, and access to resources and supports.

## Literature Review

### The Impact of Disasters on Children and Youth

Much of the research which has examined the impact of disasters on children and youth has focused on the risk factors associated with negative outcomes, such as the prevalence of anxiety, depression, PTSD, and other mental health issues (26, 27). This research largely finds that children and youth are impacted by the disruption of routine, experiences of displacement, and limited or ineffective societal or familial response to disaster (17, 18, 21). Experiences of disruption and loss contribute to feelings of stress, sadness, loneliness, and worry among children and youth populations (17, 18). While understanding the risk factors and vulnerabilities of children and youth post-disaster is necessary to address their needs, the need to develop an understanding of the protective factors and strengths they possess is equally pressing as they can serve to mediate resiliency processes following adversity or trauma, such as experiencing a disaster. Research which focuses on protective factors has shown that even in the context of adverse circumstances like disaster, when provided with the proper support systems, children and youth often demonstrate resilience (17, 19, 28).

### Resilience Among Children and Youth

Resilience, from a social-ecological perspective, is defined as “the capacity to navigate to health-enhancing resources that nurture individual, relational, and community assets, as well as the capacity of individuals to negotiate with others for these resources to be provided to them in culturally meaningful ways” (29). This understanding of resilience highlights the multiple factors that contribute to producing positive developmental outcomes among children and youth who experience adversity, including individual, family, relationship, community, and cultural factors (30, 31). Despite the vulnerability of children and youth to the impacts of disaster, recent research reveals that children and youth can and do act as powerful catalysts for recovery and change in their families, peer groups, and wider communities post-disaster (17–19, 21). A number of influencing factors have been associated with child and youth resilience, including individual, family/caregiver, peer, and larger systems.

#### Individual Factors

Individual factors refer to internal capacities which influence how children and youth respond to traumatic experiences (32). Among the many individual factors that have been examined, locus of control—that is, the degree to which an individual believes they are in control or not of the situations they find themselves in—has a strong influence on child and youth resilience (33, 34). Having strong sense of internal locus of control is associated with self-efficacy, emotional regulation, and self-control, which significantly influence attitudes toward difficult circumstances, and choice of coping strategies which are, of course, critical to children and youth building resilience in adverse contexts (33–35). Self-control enables children and youth to make decisions and respond in times of crisis (33). For example, Terranova et al. (36) found that children who were able to exert self-control in adverse situations were less likely to develop PTSD symptoms following Hurricane Katrina in 2005 in Louisiana, New Orleans.

The research also indicates that children and youth who experience disasters fare better when they are proficient problem-solvers and adept at learning (37). For example, Nelson (37) found that following Hurricane Katrina, youth who believed that they could positively respond to adverse events in their lives, or asked for help in doing so, fared much better that those who did not. Many post-disaster interventions are in fact based on providing children and youth with a greater sense of control over their environment, more opportunities to think critically and solve problems, and means to make choices for themselves (38). This is often seen as an opportunity to recognize the individual strengths that children and youth possess, and their ability to influence their families, peers, and their wider communities (39).

Having an optimistic outlook has also been associated with resilience among children and youth post-disaster (40, 41). Children and youth who view challenges as learning opportunities rather than obstacles, and are able to maintain a positive and hopeful attitude have increased resilience (21, 37). For example, Walker et al. (21) found that children who experienced the 2007 flood in Hull, England, who engaged in opportunities to reframe their situation and focus on positive outcomes through play and other home and school activities, fared better.

#### The Role of Caregivers

Having supportive relationships with one or more caregivers has also been identified to be a consistent predictor of resilience among children and youth who have experienced traumatic events, such as a disaster (13, 42). The psychological support provided by caregivers serves to protect and bolster the resilience capacities of children and youth. Having a close and supportive relationship with caregivers helps children and youth build and strengthen their resilience. Re-establishing a sense of safety, resuming normal roles and routines, and ongoing open communication with their children are some of the ways caregivers have helped children to navigate and cope with the experience of disaster (43). A number of researchers have identified that sense of family cohesiveness (44, 45), positive family communication styles (44), effective conflict resolution skills within the family (46), and related positive coping skills (47) are all associated with child and youth resiliency. For example, Hafstad et al. (43) found that children who experienced the 2004 tsunami in Southeast Asia benefitted from parents who engaged in watchful waiting, careful monitoring of reactions, and sensitive timing as ways to monitor and determine the needs of their children.

Serving as a buffer to the trauma caused by disaster, physical proximity and physical affection between caregivers and children/youth post-disaster has also been found to be an important predictor of child and youth resilience (45, 48). By spending time comforting, reassuring and caring for the physical needs of children and youth, caregivers contributed to the sense of safety and security the family experienced post-disaster. Close contact and communication enables caregivers to teach children and youth specific skills, use their strengths, and encourage open communication, all of which are essential to shoring up resiliency (32, 49).

#### Peer Influence

A more recent predictor of resilience among children and youth following experiences of adverse events such as disaster is the presence and role of peer groups (50–52). A limited but growing body of research has found that maintaining friendships during and after disaster, despite evacuation and displacement, provides children and youth with social support that is helpful to them during early recovery and ongoing rebuilding stages (14, 31). Connecting and communicating with peers helps children and youth re-establish a sense of normalcy and security, and provides mutual ongoing support and assistance among peer groups (14, 50). Peer-to-peer relationships post-disaster provide children and youth with age-appropriate connections, which encourages and facilitates the sharing of experiences and needs. Often, children and youth also rely on peers as a form of distraction from the day-to-day stressors of post-disaster life (53).

#### Schools as Support Systems

Larger systems, such as schools, can also contribute to the resiliency of children and youth who experience a traumatic event like a disaster. Given that disasters often impact a large number of children and youth in communities, post-disaster recovery efforts are often implemented within schools as a way to address the collective trauma of disaster (54). Group-based school interventions focused on routines, group problem-solving, as well as strategies directed to learning and enhancing protective factors of childhood development increase resiliency capacities (54). Schools often serve as a facilitator of recovery not only for children and youth themselves, but also for their families and the wider communities (3, 14). Within the school system, teachers and other school personnel (such as counselors and support workers) serve as a critical support systems for children and youth following disaster (14). School-based intervention programs aimed at reducing PTSD symptoms are often effective in helping children and youth recover from disaster (55). Likewise, teachers are often able to significantly reduce the post-traumatic symptoms of children and youth following disaster, thus enhancing their overall functioning within the school setting (56).

It is important to note that much of this research relies on data that is not obtained from children and youth themselves, but rather from the adults in their lives such as parents, teachers, doctors, and counselors. While information obtained from adult caregivers and allies is important, it neglects the experiences and insights of children and youth informed by their own unique perspectives which are key to understanding the factors that contribute to their resilience. In order to fully understand children and youth's resiliency post-disaster, it is crucial to include children and youth as informants, recognizing them as knowledge holders of their own experiences (19). This article aims to fill this gap in the literature by discussing findings that are based on data collected from children and youth themselves, and thus reflects their unique lived realities, experiences, perspectives, and voices.

## Methods

A mixed-methods approach was used to explore factors associated with resiliency among children and youth who experienced the 2016 Fort McMurray, Alberta wildfire. The study was conducted 1-year after the wildfire, and over a 3-month period from July to September 2017.

### Recruitment

Informed consent from parents/legal guardians and assent from child/youth participants was obtained. A total of 100 school-aged children and youth between the ages of 5- to 18-years participated in the study. The participants had a mean age of 11.01 (SD = 3.89), and slightly more than half of the participants were female (*n* = 55) compared to male (*n* = 45). All of the participants directly experienced the wildfire, and therefore faced the numerous physical, economic, psychological, and social difficulties that often occur in the aftermath of disaster.

It is important to note the large age range (5- to 18-years) of the child/youth participants. Despite variations in age differences, all of the child/youth participants demonstrated a notable recall ability and reflexive capacity. The quotes selected from the data and discussed in the qualitative findings section are drawn from participants with varying ages, gender, and community of residence, so as to represent the diverse experiences of the participants. Younger participants were more likely to discuss immediate issues and concerns, whereas older participants were more likely to move beyond current contexts and discuss long-term effects and consequences related to their disaster experience and resiliency capacities.

### Measure

The Child and Youth Resilience Measure (CYRM-28) was first administered to child and youth participants to assess factors contributing to their resilience post-disaster. Completion of the CYRM-28 ranged in length from 15 to 20 min. Informed by a social-ecological perspective, the CYRM-28 assesses three dimensions, or factors, associated with resiliency, including: (1) Individual (comprised of individual personal skills, individual peer support, and individual social skills), (2) Relationship with Primary Caregivers (caregivers physical caregiving, and caregivers psychological caregiving), and (3) Context (context spiritual, context educational, and context cultural) (57). A 5-point response scale is used for all 28 items (1 = not at all; 2 = a little; 3 = somewhat; 4 = quite a bit; 5 = a lot). Scores on the overall CYRM-28 range from 28 to 140, with higher scores reflecting higher levels of factors associated with resilience. Higher scores on each of the three CYRM-28 subscales (individual, caregivers, and contextual) also reflect higher levels of factors specific to each of these dimensions. Participants responses to the CYRM-28 measure were input, calculated, and analyzed using SPSS.

### Semi-structured Interviews

Qualitative semi-structured interviews were then conducted with child and youth participants to contextualize the CYRM-28 resiliency factors and further assess the specific ways in which individual, caregiver, and contextual factors contribute to rates of resilience among children and youth. The interview guide consisted of numerous open-ended questions which further examined children/youth's overall wildfire experience in relation to numerous factors related to resilience, such as personal skills, social skills, peer interactions, parent/caregiver availability and support, spiritual beliefs, cultural values, and educational supports. Art-based activities were utilized during the interviews to keep child/youth participants engaged, particularly younger participants. These art-based activities included clay modeling, drawing, coloring, and painting. Interviews were recorded with the participants' permission using digital voice recorders, and ranged in length from 1.5 to 2.5 h. Interviews were transcribed verbatim for analysis, and interview transcripts were uploaded and coded in NVivo. Comparisons were drawn across interviews to identify themes and meanings grounded within the data. The data were analyzed using Maxwell's (58) and Miles and Huberman's (59) qualitative analysis technique referred to as “descriptive” and “pattern” coding. Child/youth participants' responses were open-coded to identify descriptive explanations of the individual, caregiver, and contextual factors. In-depth analysis of these open categories was then conducted to identify patterned relationships across these categories and determine similarities and differences in the themes. Responses were coded and examined by the authors to ensure coding reliability. In order to interpret the coded data, we utilized Burawoy et al.'s (60) approach where arguments were constructed by creating an ongoing exchange between the data categories and existing disaster and resiliency theory in order to build upon and expand the existing theory. The quotes selected from the data and discussed in this article are drawn from child/youth participants with varying age, gender, and race/ethnicity, so as to represent diverse experiences among the participants. Ungar's (31, 57) social-ecological theory of resilience informed how we made sense of relationships between social factors that contributed to the resilience of the children and youth who participated in our study.

Ethical considerations and measures were applied throughout the study to prevent any harm to child/youth participants which may have arisen from recalling potential traumatic memories of the wildfire. A total of 6 student research assistants on the research team collected the data. These research assistants were trained in quantitative and qualitative methodologies and ethical practices prior to conducting the interviews with the child and youth participants. Parents/legal guardians of the child/youth participants were provided with a list of local counseling services, should they feel the need to access such services after the interview. Moreover, participants were given a $50 gift card as an honorarium to recognize the time they contributed in participating in the study. Ethics approval for the study was obtained from the Human Research Ethics Board at Mount Royal University. All participant names have been replaced with pseudonyms to ensure anonymity.

## Findings

I learned that we can be like, resilient. I learned that we are strong. We are strong together. That's what really cheered me up (Alonzo, 11-years old)

The findings reveal that the children and youth participants who experienced the 2016 Fort McMurray wildfire had higher than average levels of resilience. As illustrated in the quote above from one of the child/youth participants in our study, resilience is not just an individual characteristic that one is born with, but rather is a result of broader social-ecological factors such as family, peer, and community factors. The findings from our study reveal that higher levels of resilience among children and youth participants were associated with (1) caregiver factors, specifically physical caregiving; and (2) individual factors, namely peer support. We discuss below.

### Quantitative Findings: CYRM-28 Resilience Scale and Subscale Scores

Total and mean CYRM-28 scores and total and mean scores on the three CYRM-28 subscales (individual, relationship with caregivers, and contextual), and total and mean scores on subscale question clusters were calculated (see Table 1).

**Table 1:** Descriptive statistics for CYRM-28 total and subscale scores (*n* = 100).

**Name**	***N***	**Mean**	**SD**
**CYRM-28 Normative Scores (Score out of 140)**			
Total Sample	2,198	108.60	18.66
*Complex Needs Youth*	1,071	103.85	20.18
*Low-Risk Youth*	1,127	113.12	15.82
**Total CYRM-28 SCORE (Score out of 140)**			
	100	118.65	10.23
**Mean Scores for 3 Sub-Scales (Scores out of 3)**			
1) Individual	100	4.19	0.435
•Personal	100	4.15	0.476
•Peer Support	100	4.25	0.796
•Social Skills	100	4.21	0.519
2) Relationship with Primary Caregivers	100	4.41	0.418
•Physical Support	100	4.45	0.564
•Psychological Support	100	4.40	0.485
3) Context	100	4.17	0.454
•Spiritual	100	3.62	0.906
•Educational	100	4.41	0.748
•Cultural	100	4.41	0.436

The total mean CYRM-28 score was 118.65, with a standard deviation of 10.23. Normative data for the CYRM-28 with a 5-point scale indicates that for a total sample of Canadian youth the mean score is 108.60 with a standard deviation of 18.66 (for complex needs youth the mean score is 103.85 with a standard deviation of 20.18, and for low-risk youth the mean score is 113.12 with a standard deviation of 15.82). As such, the total observed mean score of 118.65 is relatively high compared to normative scores, indicating that participants had higher than average levels of characteristics associated with resilience than a normative comparison sample.

The mean scores on the three CYRM-28 sub-scales indicated that the Relationship with Primary Caregivers sub-scale had the highest overall mean (4.41) with the Physical Support cluster having the highest mean of 4.45. This indicates that participants had high levels of characteristics associated with caregiving contributing to their overall resilience, such as having their psychological needs met but especially their physical needs met by the caregivers in their life. The second highest mean was for the Individual sub-scale (4.19), with the Peer Support cluster having the highest mean of 4.25. This indicates that participants had high levels of characteristics associated with individual factors when it comes to their overall resilience, such as social skills, and especially peer support. Both Relationship with Primary Caregivers and Individual characteristics are closely associated with children and youth's resilience. The Context sub-scale was found to have the lowest overall mean (4.17), with the Spiritual cluster having the lowest mean of 3.62. This indicates that participants had low levels of characteristics associated with context contributing to their overall resilience, such as educational, cultural, and especially spiritual support.

### Qualitative Findings: Themes and Patters From Child and Youth Interviews

In-depth semi-structured qualitative interviews were conducted to contextualize participants' CYRM-28 resiliency scores and further explore the specific ways in which individual, caregiver, and contextual factors contribute to rates of resilience among child/youth participants. Given that the CYRM subscale scores indicated that high levels of resilience among child/youth participants was associated with both caregiver factors (physical support having the highest mean score), and individual factors (peer support having the highest mean score), we focus on themes and patterns described by the child/youth participants in relation to these two factors.

#### Child/Youth Resilience Post-disaster and Caregiver Support

In discussing the caregivers in their lives, children and youth largely referred to their parents. Child/youth participants discussed how their parents provided physical support during and after the wildfire by (1) ensuring their safety during the evacuation; (2) providing basic needs such as shelter, food, and clothing; and (3) offering support and reassurance by being physically present. The physical support children and youth received from their parents during and after the wildfire played an important role in their recovery, thus strengthening their resilience.

##### Ensuring Safety During Evacuation

A disaster can severely impact children and youth's sense of safety due to the suddenness of such events, which often occur without much warning or preparation. Children and youth, due to their cognitive and developmental life stage, have limited understandings about disaster events, and therefore often feel as though they have little to no control over what is happening around them, particularly during an evacuation. With the threatened destruction of the physical spaces in which they live, such as their homes, schools, and community spaces, having physical support from a caregiver is critical for ensuring both the physical and psychological security and protection of children and youth. Many child/youth participants discussed how their parents' efforts to safely and quickly evacuate their families during the wildfire helped them feel a greater sense of physical safety and security during this stressful time. For example, Lainey, an 8-year-old, discusses how being able to physically evacuate with her parents, as opposed to someone else, provided her with a sense of safety and security: “It made me feel closer because we were all sticking together, like when we were evacuating were close together and like always having plans.” Likewise, Hermione, a 14-year-old, describes how she recognized the great lengths her parents went to in order to leave their workplace and pick up both her and her siblings from their respective daycares/schools:

Just getting us out safe I think was a huge impact because I know I wouldn't— If I had been in that situation trying to get out from site, and all the kids from the daycare, and plus me and my sister, plus everything from the house, I know I would have been probably crazy. It was so stressful and they held it together really well and they got us all out safe with everything that we needed, so I think that really impacted me a lot.

Child/youth participants also discussed how during the evacuation they worried about damage to their homes, belongings, schools, recreation centers, and other infrastructures they frequently used in their communities. Many children and youth spoke about how concerned they were about potentially losing their physical belongings, such as toys, clothing, and technology (computers, tablets, and video game consoles), which added to the stress of having to evacuate their homes and communities. These were items that children and youth valued and used daily. Child/youth participants discussed how, despite these concerns, their parents continued to remain a vital source of support, reassuring them that they would take care of their physical needs by finding a place to stay, and when permitted to return home, replacing and rebuilding anything that was damaged or lost in the wildfire. For example, Francine, an 8-year-old, discusses how her parents found a safe place for them to stay during the wildfire, and worked through financial and insurance issues without her having to feel the burden or stress:

They made us feel safe by like finding us a place to stay and like, making sure we weren't worried about what was happening with like, our house or um, finances, or insurance, or anything. They took that under their belt so that we didn't have to worry about it as well. So I think like for them it was a lot harder to deal with things than for us because they didn't want us to go through that. So, I think they made it so much easier just for us in like, technical terms, not like emotionally but like, technically.

By providing physical safety and security during and post-disaster, parents helped bolster the overall health and well-being of their children, thus contributing to their recovery.

##### Providing Basic Needs

Child/youth participants frequently described the upheaval caused in their lives by displacement during and after the wildfire. Many children and youth had not anticipated that they would not be able to physically return to their homes and community for weeks, even months. As time went on, many children and youth became increasingly worried about how their basic needs would be met, including how they would access shelter, food, and clothing. Parents who took control, or sought to take control of these matters, such as planning for short- and long-term shelter for their families, ensuring their children had access to clothing and other basic necessities, helped children and youth to feel a greater sense of safety, stability, and control over the situation. For example, Veronica, an 18-year-old, discusses how she felt reassured because her parents had a plan for their family:

If we had to like, um, we weren't able to go home, my dad already had a plan to rent out a basement. Like having a plan really helped me, like I didn't feel like I was lost, like, “what's going on?” and stuff like that. Even, like I said, when we came back my dad and my mom took an active role in making sure that the house was done and getting like, insurance and stuff like that, like having the side fixed, cleaning out the house. Just they were — it really helped like, having them like, take control like — like that.

Child/youth participants also worried about the financial implications of the wildfire for their family, both short- and long-term, which created significant stress for them. Parents who provided needed assurance not only through words, but also actions, helped alleviate some of these concerns that children and youth experienced about having their physical needs met. For example, Admir, a 16-year-old, describes how he felt he could rely on his father, despite the financial challenges their family faced, because his father took action to ensure the family's basic needs were met:

My father…he was the one who went and got clothes for us and toiletries from the place where they distributed it. He was the one who talked to a lot of people, like especially our insurance about like the money that we were getting. And he was the one who worked — he's the one who works so hard for — for us.

Similarly, Franny, a 10-year-old, was concerned about the loss of her personal belongings, but reassured by her parents that they would be able to replace these items. This helped Franny gain greater perspective: “My dad would say like ‘I can buy you lots of new stuff', and I'd be like ‘It's okay.' Then my mom would say stuff like that, and then say ‘It happened to all of us, it happened to me [too]' (crying).” The effects of having a dependable caregiver reflects children and youth's need to have a source of both physical and emotional stability during times of chaos and upheaval. The presence of a caregiver who is able to provide both a structured and stable environment reduces children and youth's fears and concerns following disaster, a key factor contributing to recovery among children and youth.

##### Physical Presence and Support

Child/youth participants also discussed the importance of having their parents physically close and present during and after the wildfire. Physical proximity facilitated parents' ability to provide psychological support to their children. Many child/youth participants stated that just having their parents physically close to them, and getting to spend additional time with them during and after the wildfire was a source of comfort, and also provided them with greater perspective. Undoubtedly, losing their home and belongings presented numerous challenges to children and youth, yet many of them were quick to recognize and articulate that material things like homes can be rebuilt and belongings repurchased after a wildfire, but non-material things like families cannot be replaced. For example, Jono, a 13-year-old, explains how having his parents with him and being together as a family after the wildfire provided him with a great sense of security during an unpredictable time:

So, just the fact that they were — we were there for each other. I mean, isn't that all you need, is family? Just other people around you that you know, and that you love, and that you trust, you know? That's just — it just clicks in you. You don't really know why, it just — it makes you feel safe, secure.

Similarly, Anna, a 12-year-old female, describes how having her parents and family members physically close and spending time together helped her gain perspective about the wildfire and realize that family, and not material items, is what matters most. She states: “We all like, I guess went everywhere together. Family's like the most important thing, so if they were all okay like everything was fine. Houses can like, be rebuilt if they're burnt.” Like many others, Anna found solace in knowing her family members were safe despite the destruction caused by the wildfire.

Several child/youth participants described how the physical presence of their parents led to increased communication in the family, which helped them feel comforted and reassured. This underscores the importance of children and youth having not only their physical needs met, but also their emotional needs met through increased communication. Many child/youth participants described how communication with their parents provided a significant source of support both during and after the wildfire. Being able to speak freely about their wildfire experiences with their parents helped children and youth share their fears, concerns, and difficulties, which helped them process the event. Consequently, parents were better able to respond to the needs of their children when they were physically present, which aided in their recovery. For example, Hannah, a 15-year-old, asserts that the presence and availability of her parents provided assurance during tumultuous times: “They [parents] would always listen or always be there if I had to talk about something.” Likewise, active listening among caregivers helped support children and youth who were struggling and needed to feel heard, as 11-year-old Natalee describes: “I don't know, they just… They [parents] listened to everything that I was saying by showing that they care and not being sad for me and with me. Tried everything they can to make me happier.”

Increased communication often served as the basis for parents being able to assess where their children were at in terms of physical, as well as mental and emotional well-being. Some child/youth participants stated that they experienced mental health challenges after the wildfire, but were able to confide in their parents and receive support, which helped them to cope better. For example, 13-year-old Asher describes the emotional support his mother was able to provide him with after the wildfire:

I think my mom, because my dad like he's very helpful because he was making money working, but my mom was like our caretaker. We did a bunch of stuff and it made it easy for us to have fun and like, take our minds off of it [wildfire]. She'd support us if uh, if like, one of us was down, which was pretty good. She was calm the whole time. Like it makes you feel good (laughs). It's like the oldest one that knows more is calm then you're most likely calm.

Evident in Asher's response is the ways in which his mother actively sought to support the children in their family, as well as model open communication and positive coping skills. These findings highlight how the physical support children and youth received from their parents during and after the wildfire played a significant role in their recovery, thus strengthening their resilience.

#### Child/Youth Resilience Post-disaster and Peer Support

Individual factors, specifically peer support, is another key determinant of resilience identified by child/youth participants. Child/youth participants discussed how their peers provided support during and after the wildfire by (1) communicating with them; and (2) providing a needed distraction from immediate stressors. The support children and youth received from their peers during and after the wildfire played an important role in their recovery, thus strengthening their resilience.

##### Communication

A disaster can disrupt children and youth's social connections. Peer relationships are often important sources of stability and support for children, particularly for youth. Yet displacement during an evacuation can present challenges to peer group contact and communication, often leading to feelings of isolation and loneliness among children and youth. For many children and youth being able to interact and openly and honestly communicate, not only with their parents, but also their peers during and after the wildfire provided a valuable source of support. The opportunity to express their feelings, share their concerns, and reciprocally obtain and offer support to their peers helped children and youth better process the trauma of the wildfire. In fact, for many children and youth, communication with their peers was perceived as therapeutic. Several child/youth participants discussed how the willingness and availability of their peers to talk with them helped them feel supported. For example, Lucy, a 15-year-old, describes how her peers' commitment to talking with her whenever she felt the need made her feel cared for: “They're just like, very caring people, like by their actions and stuff. So yeah, like they always made it clear that if I needed to talk to them about anything like they were there for me.” Likewise Meredith, a 13-year-old, explains how simply knowing that her peers were available if she needed to talk helped her recover from the trauma of the wildfire:

They weren't like, off doing their own thing, like worrying about all this stuff. Like if you needed to talk to them or they needed to talk to you, like we were all kind of there for each other. It didn't matter if you were going through something, you were also there to help them get through something.

Having the opportunity to communicate with peers provided children and youth with insight into how others, similar in age and experience, were processing the wildfire and navigating both the short- and long-term challenges that result from disaster. This served to legitimize and validate their own experiences. Many child/youth participants discussed how they felt comforted by the fact that they were all going through the same thing, and that they could be open and honest about their feelings with their peers, regardless of their circumstances. For example, Hugo, an 18-year old, describes how his peers never judged him but always supported him, despite the fact that his home was not damaged by the wildfire:

There were a few times where I talked with them about it, just like what happened and all that kind of stuff, and they were always very supportive, you know. My house is fine and you know, still talking about it like it was a bad experience, there might be something [judgment] there. But nope, everyone was just like really supportive.

In addition to receiving support from their peers, children and youth also provided support to their peers, highlighting the reciprocal nature of peer support. Child/youth participants discussed how they also took an active role in supporting their peers after the wildfire, which strengthened their relationships with their peers. For example, Hannah, an 8-year-old states how her peer group supported one another when they were struggling, which served as a safety net of sorts for many of them:

They [friends] cared about me because when I was really hurt and they were really hurt I cared for them and they cared for me…When I was really scared about the fire, I was telling my friends about it, and they told me “It's okay, the fire is gone. But it can come back. Just remember what to do if it comes back.”

Likewise, Jamie, a 14-year-old, explains how she tried to create an open and non-judgmental space for her friends to reach out and share their feelings with her, which helped build and solidify their friendship:

It makes me feel good cause then it's like they will always feel like I'm there for them to talk to. Like, I feel like if they can talk to me about like anything then like, we could build like our friendship.

Other child/youth participants discussed the importance of regularly checking-in on their friends, and providing them with informal supports, whether that be just sharing their feelings, or spending time with them. For example, Sara, an 18-year-old, discusses how she helped her friends process the loss and damage of their homes and belonging, which created significant distress for many of her peers:

I did have a couple friends whose houses burnt down, but like yeah, I'm not a counselor, but like I did do my best to just talk to them, and be like, “hey like.”— I feel like you know, that helped a bit. Even just hanging out with them and stuff, and being like, “Okay, well whatever, like let's go to the mall,” you know? Things like that or like catching up or whatever. I don't know, like that's how I feel.

Openly and honestly communicating with their peers, as well as reciprocally receiving and offering support to their peers helped many children and youth in the recovery phases of the wildfire. By communicating with their peers, children and youth felt supported and heard, which validated and legitimized their unique experiences.

##### Distraction From Stressors

Child/youth participants also discussed how their peers helped provide a needed distraction from the stressors of the wildfire. Child/youth participants frequently discussed how spending time with their peers served as a useful distraction from the stresses associated with evacuation, rebuilding, and recovery. Time spent interacting with their peers, whether that be just visiting or playing/interacting with one another, provided a necessary respite from the trauma of the wildfire. For example, Kris, a 10-year-old, describes how talking on the phone with his friends after being evacuated, and then having his friends come over to his house to play once he was permitted to return to his home helped bolster his overall well-being: “During the wildfire we [friends] talked on the phone, so that made me feel better and more safe, just talking to them, hearing their voices. And after, they came to my house when I told them I came home right away and then we played.” Similarly, Margaery, a 15-year-old, shares how the willingness of her peers to talk with her through text, or meet up in-person to discuss even mundane things provided a useful, temporary escape from the negative effects of the wildfire:

They checked up on me, you know? They'd ask what was going on or like again, if I texted them and said like, “Hey, this happened.” They'd be interested in it and they'd let me talk…or they'd be willing to meet up with me if I wanted to, if we could.

For some child/youth participants, spending time with their peers after the wildfire boosted their morale, and helped them regain a sense of happiness despite the challenges they continued to face in the rebuilding and recovery stages. Catherine, a 7-year-old, discusses how just spending time with her friends brought her a sense of joy, which in turn helped her to forget about the stressors of experiencing the wildfire: “My friends helped me because they like made me feel good about myself. When I'm with them [talking], they make me feel like happy, which helps me forget about the wildfire and helps me not to worry.” Having their peers distract them helped many children and youth cope with the stressors of the wildfire. This reflects children and youth's need to sometimes take a step back from adverse situations to feel a sense of normalcy again, even if only temporarily.

Child/youth participants also discussed how their peers helped distract them from the negative impacts of the wildfire by helping change their mindset. Many child/youth participants expressed that their peers helped them focus on some of the positive outcomes that emerged from the wildfire. For example, Franny, a 10-year-old, states that her peers tried to cheer her up by reminding her of the new items she received at school which were bought to replace the ones that were damaged: “They would say stuff like, ‘Look at all the gifts you get at school now, like you're really lucky,' and stuff like that. So, you know, that would make me feel better.” Similarly, Allison, a 16-year-old, discusses how her peers helped take her mind off of the extensive damage in their community by sharing the new items they received, but also sharing humorous and amusing stories and experiences: “They just like kept my mind off of it. We were always talking about, ‘Oh look, I got this new stuff, and I got this new stuff' (laughs). I'd tell them stories and jokes and whatever, we were just talking about random things that were happening.” Humor was something that child/youth participants frequently cited as one way that their peers boosted their mood.

Some child/youth participants even shared that their peers helped distract them by recommending ways to keep themselves busy, like books to read, games to play, television series to watch, among others. For example, Astrid, a 12-year-old explains how his friends lightened his mood with their humor, but also by recommending a new book series which helped keep him busy:

Most of my friends are a lot of jokers. So, I think they were making puns about it all throughout (laughs). So, that really lifted my spirits. I had a friend who got me into this like, series. I can't even remember what it was called. But this friend got me into this series, and I read that in like, a week (laughs). I guess, again, just being there was most of the big part.

Evident in Astrid's response is the various ways in which his peers actively sought to uplift him by helping him have a more positive mindset and encouraging him to pursue activities that brought him a sense of joy. These findings highlight how the support children and youth received from their peers during and after the wildfire played a significant role in their recovery, thus strengthening their resilience.

## Discussion

### Quantitative Findings

The quantitative findings from the CYRM-28 measure revealed that child/youth participants who experienced the 2016 Fort McMurray wildfire had higher than average resilience scores compared to normative comparison samples. These findings indicate that wildfire-affected child/youth participants had significant strengths, capabilities, and supports at their disposal in the aftermath of the wildfire, which helped them in the face of adversity.

The findings revealed that child/youth participants with higher resilience scores had higher scores on the subscales Relationship with Primary Caregivers (specifically caregivers physical caregiving) and Individual (specifically individual peer support). These findings reveal that caregiver physical support and individual peer support were the most influential factors that contributed to the resiliency of wildfire-affected child/youth participants. It is important to note that the CYRM-28 measure of caregiver physical support relates almost exclusively to the behavior of caregivers (supervision provided by caregivers, and basic needs such as food provided by caregivers), whereas the measure of individual peer support relates not only to the behavior of peers, but also to the individual's perception of peer support (the presence of peers during challenges, and feeling supported by peers). Therefore, there is a possibility that children/youth's individual perception of support from peers is as important as actually having support from peers when it comes to resilience^1^.

Following disaster, children and youth experience disruption and instability in their lives as a result of the destruction or loss of physical structures and belongings which they rely on for their everyday functioning. Yet, having physical support from caregivers as well as individual support from peers post-disaster are important determinants that can mitigate the disruption and instability caused by disaster, thus contributing to higher levels of resilience among children and youth. These findings point to the importance and necessity of ensuring that children and youth's basic physical and peer needs are met post-disaster, in order to increase their resiliency and recovery.

### Qualitative Findings

#### Child/Youth Resilience Post-disaster and Caregiver Support

The qualitative findings revealed that child/youth participants with higher levels of resilience had parents who provided physical support during and after the wildfire. Parents provided child/youth participants with physical support during and after the wildfire by (1) ensuring their safety during the evacuation; (2) providing basic needs such as shelter, food, and clothing; and (3) offering support and reassurance by being physically present. These findings illustrate that caregiver support, specifically physical support, during and after the wildfire was a significant source of support for child/youth participants and helped strengthen their resilience. The findings of this study are consistent with previous research which indicates the benefits of children and youth being physically and emotionally close to their caregivers following disaster (48, 61, 62). Children and youth are better able to build and strengthen their resilience when they have strong support systems that provide a sense of security and normalcy in their lives. Similar to Botey and Kulig's (45) findings, our findings reveal that children and youth cope better following disaster when parents attempt to provide stability for their children by focusing on their physical needs and attempting to provide a sense of normalcy. Similar to Hackbarth et al.'s (61) findings, our findings also support the link between physical support in terms of having basic needs met or sought after by caregivers, rather than material belongings, as a key factor in children and youth's ability to cope post-disaster.

Furthermore, our findings reveal that when parents are in close proximity to their children, they are able to not only offer physical support, but also psychological support due to their increased presence and availability. Similar to Salloum and Lewis' (47) findings, our findings also reveal that emotional processing, a common coping strategy used within the parent-child dynamic following disaster, helps children recover as it provides them with the opportunity to share and validate their unique experiences, as well as forge stronger bonds with their caregivers. These findings suggest that parental recognition of the care their children needed may play as important of a role as the actual care given to children. The findings of this study highlight the crucial role that physical caregiving plays in strengthening children and youth's resilience post-disaster.

#### Child/Youth Resilience Post-disaster and Peer Support

The qualitative findings also revealed that child/youth participants with higher levels of resilience had peers who provided support during and after the wildfire. Peers provided child/youth participants with support by (1) communicating with them; and (2) providing a needed distraction from immediate stressors. These findings illustrate that individual factors, specifically peer support, during and after the wildfire was a significant source of support for child/youth participants and helped strengthen their resilience. The findings of this study lend support to previous research which finds that communication and comfort among peers is a significant source of support for many children and youth post-disaster, thus strengthening their resilience (19, 50). Similar to Prinstein et al.'s (50) findings, our findings reveal that peers can be among the most critical factors in helping children and youth cope post-disaster. By interacting with their peers, children and youth were able to undergo emotional processing and gain insight into the ways in which their peers were coping in the aftermath of the wildfire. While children understood the damages caused by the wildfire, they also kept a positive attitude which was reflected in the way in which peers encouraged each other to reflect and focus on the positive outcomes of the wildfire.

In addition, the findings of this study also remain consistent with previous research which indicates that peers play an important role in supporting children and youth post-disaster by helping them re-establish a sense of routine and normalcy (53). Similar to Fothergill and Peek's (14) findings, our findings indicate that spending time with peers serves as a useful distraction from the post-disaster challenges associated with rebuilding and recovery, helping children and youth better cope with the adversity of disaster. Children and youth displayed incredible agency and emotional awareness by recognizing when their friends needed to be distracted from wildfire-related worries. The findings of this study underscore how children and youth are capable of providing useful sources of support not only to their peer groups, but also their wider communities, thus strengthening individual and community resilience in the aftermath of disaster.

## Conclusion

The current study was conducted following the 2016 Fort McMurray, Alberta wildfire in order to examine the factors associated with high levels of resilience among children and youth post-disaster. This study utilized a mixed-methods approach, which involved administering the CYRM-28 resilience measure and face-to-face qualitative interviews with 100 children and youth between the ages of 5- and 18-years. This study found that despite the various challenges that children and youth experienced as a result of the wildfire, they had higher than average levels of resilience. The findings reveal that high levels of resilience among children and youth are associated with caregiver factors, namely caregiver physical support, as well as individual factors, specifically peer support.

The findings of this study build on and contribute to empirical knowledge and evidence related to the social-ecological factors that enhance resilience in children and youth who experience disaster. These findings demonstrate that when supported, children and youth are better able to cope with the negative effects of disaster, thus bolstering their resilience. These findings also support the need for additional research which takes a strength-based approach in order to unearth the protective factors that contribute to resilience among disaster-affected children and youth. Research, policy, and practice that recognizes the specific strengths and capacities of children and youth in the aftermath of disaster may facilitate the development and implementation of programs, services, and resources that can help children and youth learn and develop resilience skills. Understanding the determinant factors that contribute to high levels of resilience among children and youth is critical due to the increasing frequency and occurrence of disasters and other catastrophic events like the current COVID-19 health pandemic.

## Data Availability Statement

The original contributions presented in the study are included in the article/supplementary material, further inquiries can be directed to the corresponding author/s.

## Ethics Statement

The studies involving human participants were reviewed and approved by Human Research Ethics Board, Mount Royal University. Written informed consent to participate in this study was provided by the participants' legal guardian/next of kin.

## Author Contributions

CM-H, JD, MB, and PS: study design. CM-H: data collection. CM-H, JD, and AS: analysis. CM-H, JD, AS, MB, PS, PB-M, and VA: manuscript preparation. All authors contributed to the article and approved the submitted version.

## Conflict of Interest

The authors declare that the research was conducted in the absence of any commercial or financial relationships that could be construed as a potential conflict of interest. The reviewer SD declared a shared affiliation, with no collaboration, with the authors to the handling editor at the time of the review.

## Publisher's Note

All claims expressed in this article are solely those of the authors and do not necessarily represent those of their affiliated organizations, or those of the publisher, the editors and the reviewers. Any product that may be evaluated in this article, or claim that may be made by its manufacturer, is not guaranteed or endorsed by the publisher.

## Footnotes

^1^In the CYRM-28, the two questions in the sub-scale Caregiver: Physical Care Giving that measure caregiver physical support are: “My parent(s)/caregiver(s) watch me closely,” and “If I am hungry, there is enough to eat.” The two questions in the sub-scale “Individual: Individual Peer Support” that measure peer support are: “I feel supported by my friends,” and “My friends stand by me during difficult times” (57).
